# *Helichrysum* Genus and Compound Activities in the Management of Diabetes Mellitus

**DOI:** 10.3390/plants11101386

**Published:** 2022-05-23

**Authors:** Akeem O. Akinfenwa, Idowu J. Sagbo, Masixole Makhaba, Wilfred T. Mabusela, Ahmed A. Hussein

**Affiliations:** 1 Chemistry Department, Cape Peninsula University of Technology, Symphony Road, Bellville Campus, Bellville 7535, South Africa; 217305296@mycput.ac.za; 2 Chemistry Department, University of the Western Cape, Private Bag X17, Bellville 7535, South Africa; 3241470@myuwc.ac.za (M.M.); wmabusela@uwc.ac.za (W.T.M.)

**Keywords:** antidiabetic drugs, *Helichrysum* species, medicinal plants, diabetes mellitus, compounds

## Abstract

The global management of diabetes mellitus (DM) involves the administration of recommended anti-diabetic drugs in addition to a non-sedentary lifestyle upon diagnosis. Despite the success recorded from these synthetic drugs, the traditional method of treatment using medicinal plants is increasingly accepted by the locals due to its low cost and the perceived no side effects. *Helichrysum* species are used in folk medicine and are documented for the treatment of DM in different regions of the world. This study reviews *Helichrysum* species and its compounds’ activities in the management of DM. An extensive literature search was carried out, utilizing several scientific databases, ethnobotanical books, theses, and dissertations. About twenty-two *Helichrysum* species were reported for the treatment of diabetes in different regions of the world. Among these *Helichrysum* species, only fifteen have been scientifically investigated for their antidiabetic activities, and twelve compounds were identified as bioactive constituents for diabetes. This present review study will be a useful tool for scientists and health professionals working in the field of pharmacology and therapeutics to develop potent antidiabetic drugs that are devoid of side effects.

## 1. Introduction

Diabetes mellitus is a very prevalent disease affecting both developed and developing countries. Concerted efforts by the International Diabetes Federation (IDF) and the American Diabetes Association (ADA) to reduce the spike in global diabetes cases and mortality have witnessed different advocacies over the past years. The IDF [1] report shows that 463 million (9.3%) adults worldwide are suffering from diabetes, and this number is projected to increase by 51% in 2030 (578 million) and 2045 (700 million). The prevalence of diabetes varies according to geographical region, with more than 80% of diabetic patients living in low-to-middle-income countries, which poses additional challenges with ineffective treatment [2]. Diabetes mellitus is caused by increased blood glucose levels (hyperglycemia) due to defects in insulin action, insulin secretion, or both [3].

The two common types of diabetes (insulin-dependent (type 1) and non-insulin-dependent (type 2)) occur when the body cannot properly store and use glucose. Type 1 is reported to be common in children and is controlled by an autoimmune disorder resulting in a lack of insulin production. Type 2 diabetes is prevalent among adults, characterized by insufficient insulin production and or sensitivity to glucose uptake [4]. Both types of diabetes can lead to life-threatening complications such as neurological conditions, cardiovascular disease, damage to blood vessels, kidney disease, and vision loss [5]. In general, the common symptoms of diabetes mellitus include increased hunger, numbness in hands and feet, frequent urination, excessive thirst, tiredness and fatigue, blurred vision, sores that take long to heal, and sexual dysfunction in men [6]. To date, there is no cure for diabetes; however, several efforts including the use of medicinal plants such as *Helichrysum* species are continuously targeted to find a permanent treatment for diabetes.

### 1.1. Conventional Treatment of Diabetes

**Figure 1:**
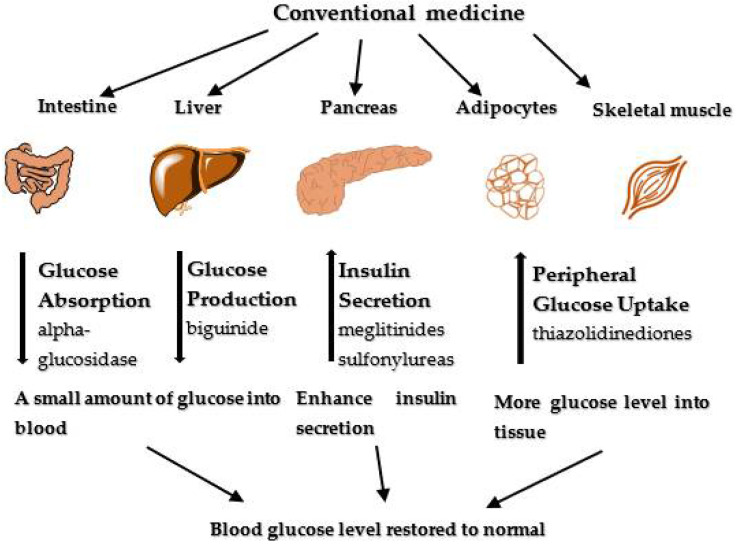
Several action sites of conventional medicines (current antidiabetic drugs).

The conventional treatment of diabetes requires oral administration of synthetic hypoglycemic agents. These synthetic hypoglycemic agents include alpha-glucosidase inhibitors (acarbose and miglitol), insulin secretagogues (meglitinides and sulfonylureas), and insulin sensitizers (thiazolidinediones, biguanides, and metformin), among others that ultimately suppress increasing plasma glucose levels (Figure 1). These drugs are known to function in two distinct ways: (1) during glucose synthesis via enzyme inhibition of glycopolymers breakdown and, (2) insulin bioavailability via the repair of β-cells of the pancreas, thereby improving insulin release and sensitivity for glucose uptake [7]. However, despite the use of these glucose-lowering drugs for the treatment of diabetes, most of these drugs have been reported with negative side effects, such as abdominal pain, headache, dizziness, diarrhea, flatulence, and digestive discomfort [8,9]. In addition, the high cost of these drugs also limits their usage. Hence, there is a need for a cheaper and more efficient drug through the application of natural products from medicinal plants with near-zero side effects [10].

### 1.2. Medicinal Plants as Alternative Therapies for Diabetes

In line with some drawbacks linked with the use of current glucose-lowering (antidiabetic) drugs, medicinal plants have been reported to play a significant role and serve as alternative therapies for the treatment of diabetes mellitus [11,12,13]. This is mostly due to the presence of several antidiabetic compounds (alkaloid, phenolic, flavonoid, and tannin), thereby improving the ability of pancreatic tissues to enhance insulin secretion or reducing the intestinal absorption of glucose [14]. In addition, the least side effects, ease of availability, and lower cost also make medicinal plants the main key players in the treatment of diabetes. Recently, the number of people with diabetes cases has been growing steadily and causing increasing concerns in most developing countries. Despite the presence of several antidiabetic drugs in the pharmaceutical market, the treatment of diabetes using medicinal plants has been recommended and often successful [15]. In the literature, various research areas have reported the use of medicinal plants and their active components as alternative sources for the treatment of diabetes [16,17]. For example, Salehi et al. [18] reported the antidiabetic of medicinal plants and their active compounds. In the study, the authors described several medicinal plants with anti-diabetic potential. Duarte et al. [19] also reported naturally occurring compounds from different plant extracts exhibiting inhibition of alpha-amylase, alpha-glucosidase, and related enzymes in the management of type II diabetes. Another study by IfedibaluChukwu et al. [20] reported in vivo antidiabetic properties of isolated compounds from the methanol stem bark extract of *Vernonia amygdalina* using streptozotocin-induced diabetes rats. In the study, it was revealed that the isolated compound (6β, 10β, 14β-trimethylheptadecan-15α-olyl-15-*O*-β-d-glucopyranosyl-1,5β-olide) demonstrated a significant reduction in the blood glucose as compared to standard metformin. Studies conducted by Hasan et al. [21] reviewed a list of medicinal plants and their compounds with proven antidiabetic activities in vivo and in vitro. The antidiabetic properties of these reported plants are often attributed to their different phytochemical constituents [22]. Interestingly, these phytochemicals are well distributed in many species, including the *Helichrysum* species used in folk medicine for the management of diabetes. However, there are inadequate studies reporting on *Helichrysum* species and their compounds used for the treatment of diabetes. Considering the traditional use of *Helichrysum* species in many parts of the world for the treatment of diabetes, the current study was undertaken to review the *Helichrysum* genus on species used in the management of diabetes and identify the bioactive constituents with reported antidiabetic activities. This review study is expected to identify the present knowledge gap and provide an important baseline for future studies.

## 2. Results and Discussions

### 2.1. An Overview of Ethnobotanical and Pharmacological Relevance of Helichrysum Genus

The *Helichrysum* genus encompasses typically aromatic herbs and shrubs with dense leaves that belong to the family of Asteraceae. The genus is widely distributed worldwide but is mostly found in Africa, with its highest diversity in South Africa, where about 500 known species occur. The plants belonging to this genus are well-known as everlasting flowers with leaves oblong to lanceolate. They have been in use for more than 3000 years for various folkloric purposes [23]. In traditional medicine, some *Helichrysum* plant parts are either drunk as teas or prepared as “burnt offering” smoke to appeal for blessings from the ancestors and are used to purify the home of the sick patients [24]. In addition, the plant from the *Helichrysum* genus has also been reported in traditional medicine for the treatment of several ailments, including stomach pain, gall bladder problems, jaundice, colds, wound healing, diabetes mellitus, skin infections, and asthma [25,26,27]. Nevertheless, with the emergence of scientific data on the use of *Helichrysum* species in the last few decades, some of the reported traditional claims have been scientifically supported. To mention a few, Tirillini et al. [28] reported the antioxidant activity of methanol extract of *Helichrysum foetidum* from east Africa. Additionally, research conducted by Matić et al. [29] revealed the antitumor potential of *Helichrysum zivojinii* extract. Another study conducted by Süzgeç-Selçuk and Birteksöz [30] reported the antimicrobial actions of flavonoids isolated from *Helichrysum chasmolycicum*. Ranaivoarisoa et al. [31] also reported the anti-plasmodial effect of *Helichrysum gymnocephalum* from Southern Africa. The anti-inflammatory activity of *Helichrysum stoechas* extracts from north Africa has also been reported [32] among others. It is imperative to note that several plants belonging to the *Helichrysum* genus have been more extensively researched for various bioactivities than their role as antidiabetic agents.

### 2.2. Antidiabetic Potentials of Helichrysum Species and Metabolites in Folk Medicine

**Figure 2:**
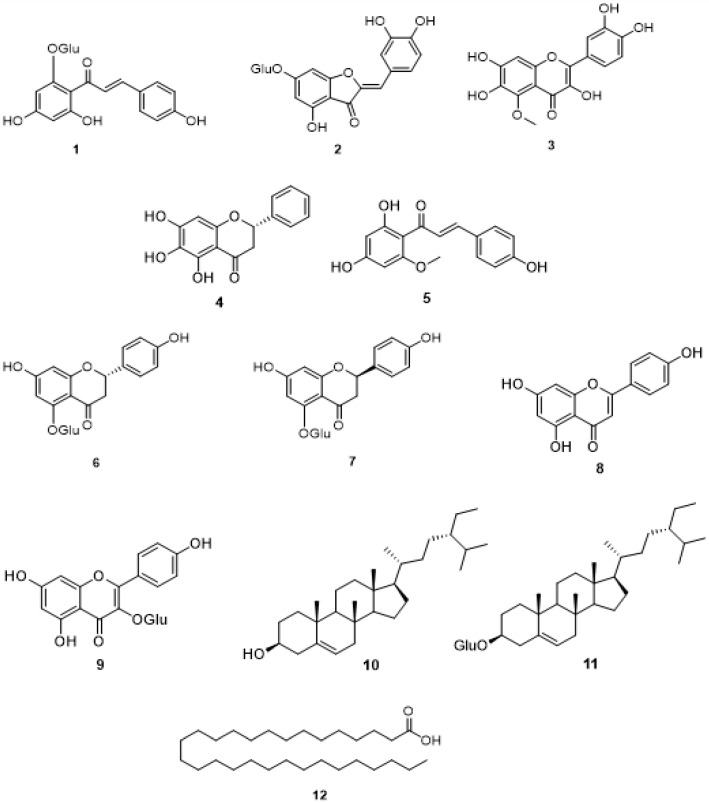
Secondary metabolites isolated from *Helichrysum* species with antidiabetic activity. The numbers **1**–**12** correspond to the compounds reported in Table 2.

Several *Helichrysum* species used for the treatment of diabetes have been identified in the literature (Table 1). Despite this, not all have been scientifically investigated for their antidiabetic activity (Table 2). In addition, only a few compounds obtained from these *Helichrysum* species have been shown to exhibit antidiabetic activity (Figure 2). Thus, in this section, a comprehensive description of plant species belonging to the genus *Helichrysum* used in the management of diabetes along with the compounds displaying antidiabetic activity will be elaborated.

#### 2.2.1. *Helichrysum arenarium*

##### Description and Ethnobotanical Usage

**Figure 3:**
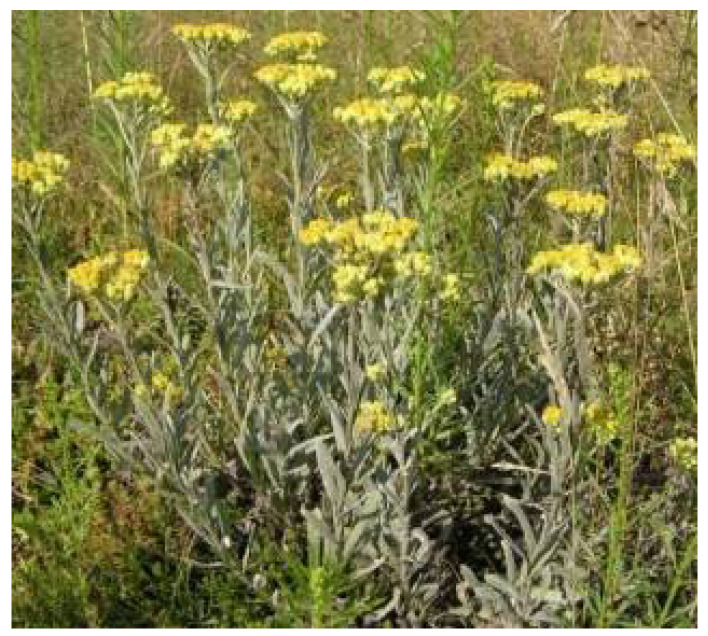
*H. arenarium*. Source: POWO [35].

*Helichrysum arenarium* (Figure 3) is a perennial herb that grows up to 50 cm in height with a robust and short rhizome [33]. The stem of the plant is generally branched at the upper part and carries alternate leaves of about 2 to 5 cm in length. *H. arenarium* is widely dispersed in Europe, Central Asia, and China [33]. The plant is well known in traditional medicine. The decoction from the aerial parts of *H. arenarium* is used for the treatment of diabetes [33]. The flowers are also reported to contain constituents and bitter substances used to promote gastric and pancreatic secretion. In addition, the infusions of the *H. arenarium* inflorescence are also used in the treatment of gallbladder disorders (rheumatism, cystitis, gout, arthritis) [34].

##### Toxicity 

Kramberger et al. [36] documented the toxicity of *H. arenarium*. In the study, the authors revealed that the aqueous extract was only toxic at the highest concentration (5%, *v*/*v*) against lymphoma (U937) cells, while the same extract displayed toxicity even at 1% (*v*/*v*) concentration in both the human colorectal adenocarcinoma (Caco-2) and primary colon fibroblast (CCD112CoN) cell lines.

##### In Vitro Antidiabetic Study

Research conducted by Morikawa et al. [37] showed that the methanol extract of *H. arenarium* inhibited the dipeptidyl peptidase-IV (DPP4) activity with an IC_50_ value of 41.2 µg/mL.

##### In Vivo Antidiabetic Study

The in vivo antidiabetic studies conducted by Morikawa et al. [37] also revealed that the extract showed significant inhibition against the increase in blood glucose levels in sucrose-loaded mice at a concentration of 500 mg/kg.

##### Antidiabetic Activity of Isolated Compounds

Two compounds, chalconaringenin 2′-*O*-β-D-glucopyranoside (isosalipurposide, 1) and aureusidin 6-*O*-β-d-glucopyranoside (2), obtained from the methanol flower extract of *H. arenarium* have been reported to exhibit strong inhibition against DPP4 enzyme activity, with IC_50_ values of 23.1 and 24.3 μM, respectively [37]. The percentage composition of compounds **1** and **2** was reported to be 0.013% and 0.0025%, respectively [37].

#### 2.2.2. *Helichrysum aureum*

##### Description and Ethnobotanical Usage

**Figure 4:**
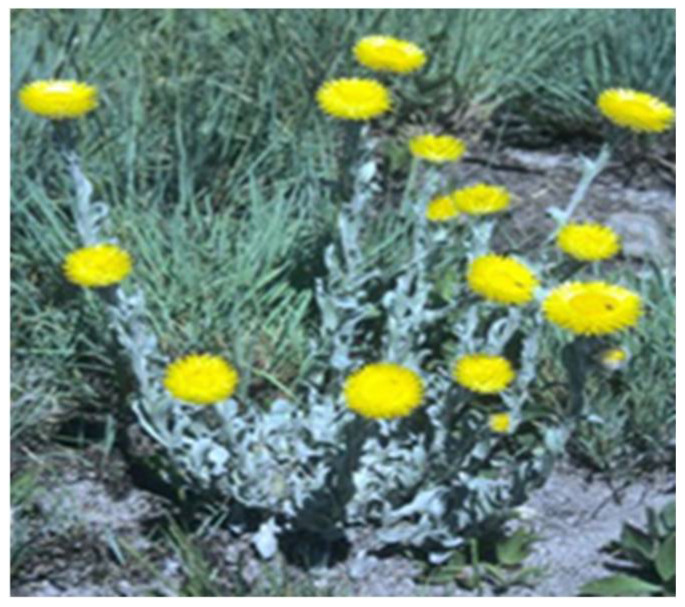
*H. aureum*. Source: SANBI [39].

*Helichrysum aureum* (Figure 4) is a perennial plant with a woody rootstock and rosette of radical leaves. It has flowering stems of 0.1–0.6 m in height with small leaves. *H. aureum* is native to Swaziland, Zimbabwe, Angola, South Africa, and Mozambique. In South Africa, it is broadly dispersed in the Cape provinces, KwaZulu-Natal, and Free State. Traditionally, *H. aureum* is used by the people of Basotho for the treatment of diabetes [38].

##### Toxicity

The cytotoxicity study reported by Lourens et al. [40] revealed that the chloroform:methanol (1:1) extract of *H. aureum* displayed cytotoxic effects toward transformed human kidney epithelial (Graham) cells, breast adenocarcinoma (MCF-7), and glioblastoma (SF-268) cells at the tested concentration (0.1 mg/mL) with inhibition of 5%, 7%, and 35%, respectively.

##### In Vitro Antidiabetic Study

The literature surveys revealed no reported scientific validation of the in vitro antidiabetic activity of *H. aureum*.

##### In Vivo Antidiabetic Study

To date, there are no reported in vivo studies of any extracts from *H. aureum*.

##### Antidiabetic Activity of Isolated Compounds

Literature survey revealed no reports on the antidiabetic activity of compounds from *H. aureum*.

#### 2.2.3. *Helichrysum caespititium*

##### Description and Ethnobotanical Usage

**Figure 5:**
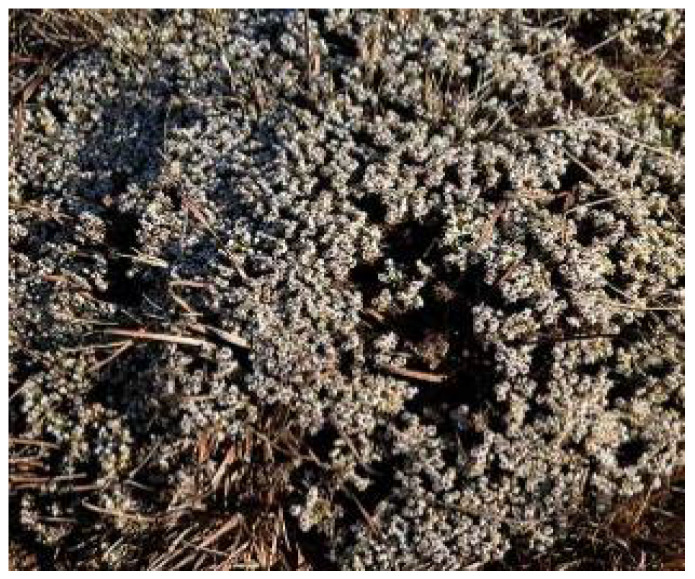
*H. caespititium*. Source: Flora of Zimbabwe [44].

*Helichrysum caespititium* (Figure 5) is a perennial creeping plant of 10 to 20 cm in height. The leaves of the plant are linear, clutching at the base and hairy on both sides, while its flowers are white to yellow [41]. *H. caespititium* is broadly distributed in Lesotho, Zimbabwe, South Africa, and Swaziland [42]. In South Africa, the whole plant of *H. caespititium* is cooked and then used to alleviate diabetes mellitus [43]. Additionally, the plant is also used for the treatment of some medical conditions such as wounds, ulceration, skin infection diseases, nausea, tuberculosis, bronco-pneumonia, and sexually transmitted infections [27].

##### Toxicity

Research conducted by Mamabolo et al. [45] reported the toxicity of *H. caespititium*. The findings of the study showed that the whole plant extracts (hexane, dichloromethane, methanol, and aqueous extracts) of *H. caespititium* had low-to-high toxic effects in rat hepatoma (H411E) cell lines. In the study, the highest toxicity was reported for the dichloromethane whole plant extract of *H. caespititium* with a lethal concentration 50 (LC_50_) value of 82.86 μg/mL compared to the standard control, doxorubicin (LC_50_ = 10.80 μg/mL).

##### In Vitro Antidiabetic Study

It is imperative to note that the in vitro antidiabetic activity of *H. caespititium* has not been scientifically investigated.

##### In Vivo Antidiabetic Study

To date, there has been no report on the in vivo antidiabetic activity of *H. caespititium* in the literature.

##### Antidiabetic Activity of Isolated Compounds

Presently, information on the antidiabetic activity of isolated compound from *H. caespititium* is very scanty in the literature.

#### 2.2.4. *Helichrysum graveolens*

##### Description and Ethnobotanical Usage

**Figure 6:**
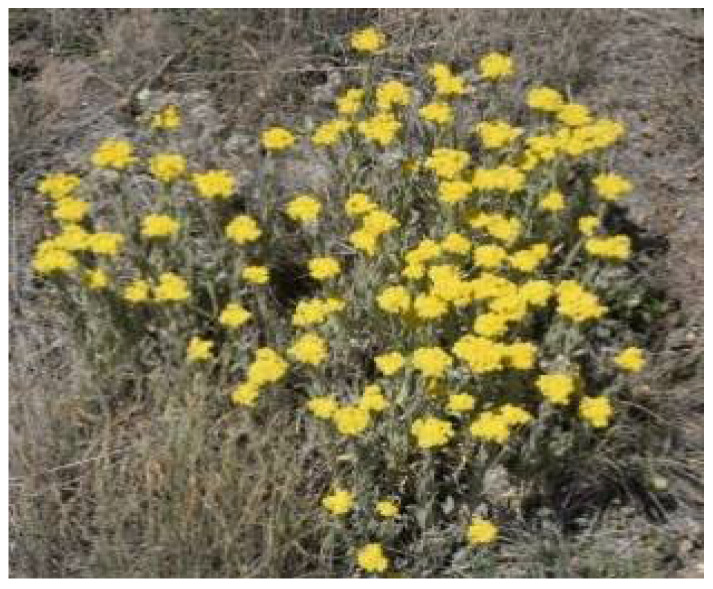
*H. graveolens*. Source: POWO [47].

*Helichrysum graveolens* (Figure 6) is an herbaceous plant belonging to the *Helichrysum* genus, with grey-bushy foliage and thin everlasting flower-heads. The plant is native to Eastern Europe, Caucasus, Turkey, Iran, and South Africa [46]. Traditionally, the decoction from *H. graveolens* has been reported to be active in the treatment of diabetes mellitus in several regions of South Africa, Anatolia, and Turkey [46]. The capitulums of the plant are also reported to be consumed for the treatment of jaundice, diuretic, and wound healing in the rural districts of Anatolia [46].

##### Toxicity

Studies on the toxicity of *H. graveolens* revealed no toxicity activity displayed by the plant against the tested cells [48,49]. Kutluk et al. [48] investigated and reported that the whole plant aqueous and ethanol extracts were not toxic to Vero African green monkey kidney cell lines, even at the highest tested concentration of 64 µg/mL. Yazdi et al. [49] supported these results, whereby they reported no toxicity effects of the aerial parts aqueous extract of *H. graveolens* in C26 colon carcinoma cells up to 5.0 µg/mL concentration.

##### In Vitro Antidiabetic Study

Several reports have confirmed the antidiabetic activities of *H. graveolens*. Orhan et al. [50] revealed that the hydroethanolic extract from *H. graveolens* exhibited 55.7% inhibition at a concentration of 3000 µg/mL against alpha-amylase enzyme. In the same study, the authors also showed that the same extract demonstrated significant inhibition against the alpha-glucosidase enzyme with IC_50_ values of 0.7129 mg/mL.

##### In Vivo Antidiabetic Study

A study by Aslan et al. [46] showed that the aqueous and ethanol extracts of *H. graveolens* significantly reduced blood glucose levels in streptozotocin-induced diabetic rats at a 500 mg/kg dose concentration.

##### Antidiabetic Activity of Isolated Compounds

A literature search revealed no report of antidiabetic compounds from *H. graveolens*.

#### 2.2.5. *Helichrysum gymnocomum*

##### Description and Ethnobotanical Usage

**Figure 7:**
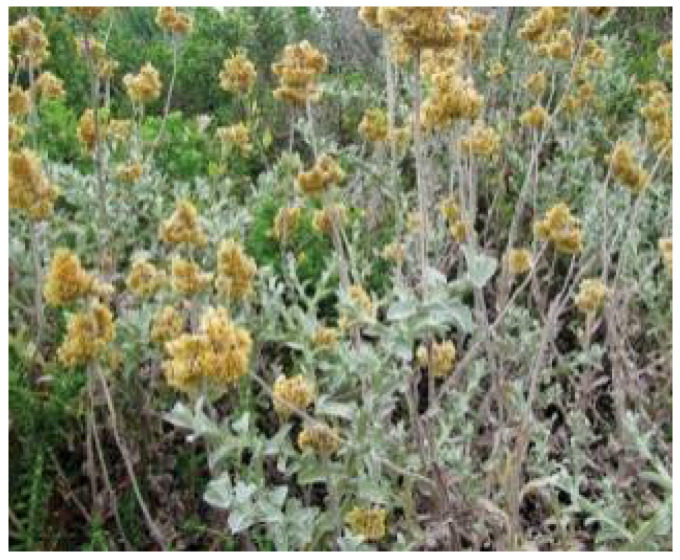
*H. gymnocomum*. Source: FOSTER [53].

*Helichrysum gymnocomum* (Figure 7) is a straggling aromatic perennial herb with pleasantly scented flowers. The stems of the plant are often decumbent and rooting at the base while the leaves are very variable and pleasantly scented [24]. *H. gymnocomum* grows abundantly in the Eastern Cape and KwaZulu-Natal provinces of South Africa [51]. In addition, the plant is also native to Lesotho. Traditionally, the decoction of the fresh leaves of the plant is taken orally for the treatment of diabetes [52].

##### Toxicity 

An extensive search of the literature at the time of compiling this review revealed no scientific report on the toxicity activity of *H. gymnocomum*.

##### In Vitro Antidiabetic Study

No reported in vitro studies were found in the literature.

##### In vivo antidiabetic study

No reported in vivo studies were found in the literature.

##### Antidiabetic activity of isolated compounds

Bioactive constituents in diabetes from *H. gymnocomum* are yet to be reported.

#### 2.2.6. *Helichrysum italicum*

##### Description and Ethnobotanical Usage

**Figure 8:**
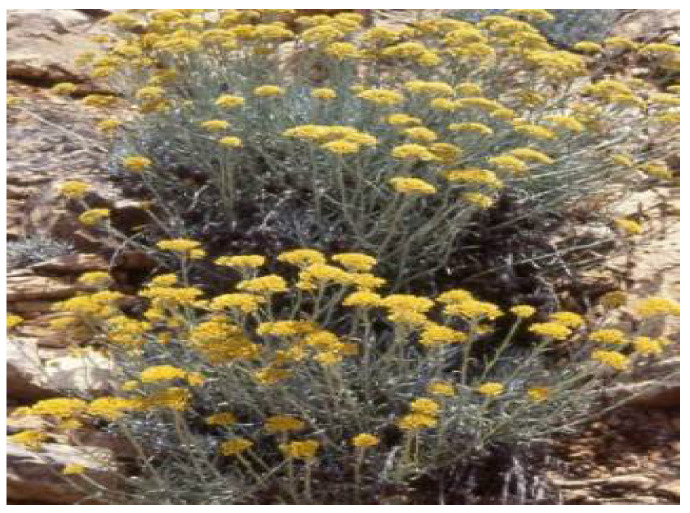
*H. italicum*. Source: American Botanical Council [56].

*Helichrysum italicum* (Figure 8) is a small evergreen shrub that grows on dry, rocky, and sandy ground. It has small leaves with a revolute margin and woody stems at the base and is 60 cm or more in height. *H. italicum* is native to Mediterranean countries such as Turkey, Portugal, Italy, and Greece [54]. The infusion or decoction of the plant is traditionally used for the treatment of diabetes [55]. In addition, infusion and decoction are also used to treat dermatologic, digestive, and respiratory disorders.

##### Toxicity

Toxicity studies involving *H. italicum* have mainly been concerted in vitro [57]. Kramberger et al. [36] evaluated cell viability on lymphoma cell line (U937), adenocarcinoma cell line (Caco-2), and primary colon fibroblasts (CCD112CoN) after exposure to the aerial parts infusion of *H. italicum*. The study reported that the infusion was not toxic up to 5% *v*/*v* concentration in U937 cells, whereas for Caco-2 it was toxic at 1% *v*/*v*. A higher concentration (2% *v*/*v*) was toxic for CCD112CoN cells than for cancerous cell line Caco-2. Staver et al. [58] and Gismondi et al. [59] independently showed that *H. italicum* essential oil exhibited toxicity effects against HeLa human cervix adenocarcinoma (IC_50_ = 0.075 mg/mL) and MCF-7 human breast cancer (IC_50_ = 0.057 mg/mL) cells, as well as B16F10 murine melanoma, respectively, in a dose-dependent manner. Nostro et al. [60], in their research study assessing the genotoxicity of *H. italicum*, found that the diethyl ether extract of the plant exhibited no DNA damaging activity, even at the highest concentration (2000 g/disc).

##### In Vitro Antidiabetic Study

Research on the in vitro antidiabetic activity of *H. italicum* has been investigated [55,61,62]. The study conducted by Pereira et al. [55] revealed that the water-based preparation (infusion and decoction) from *H. italicum* flowers exhibited moderate inhibition of alpha-glucosidase activity compared to the control at 10 mg/mL. In the research study by de la Garza et al. [61], the methanol:water (1:1) extract of *H. italicum* was reported to exhibit significant inhibitory activity against both alpha-glucosidase and alpha-amylase enzymes, with IC_50_ values of 0.19 and 0.83 mg/mL, respectively. Aćimović et al. [62], in their research study, showed that *H. italicum* essential oil had strong inhibitory activity on alpha-glucosidase enzyme (62.02%) at the tested concentration (250 mg/mL).

##### In Vivo Antidiabetic Study

The in vivo study reported by de la Garza et al. [61] demonstrated that *H. italicum* methanol:water (1:1) extract reduced blood glucose levels, thereby improving postprandial glycemic control in rats. In a separate study [63], it was shown by the authors that the methanol:water (3:1) extract of *H. italicum* ameliorated hyperglycaemia in db/db mice. Another research study [64] revealed that the methanol:water (3:1) extract of *H. italicum* markedly reduced hyperinsulinemia and insulin resistance induced by high-fat sucrose (HFS) diet in insulin-resistant rats (at 2 g/kg concentration).

##### Antidiabetic Activity of Isolated Compounds

Presently, no studies have been reported on the antidiabetic activity of isolated compounds from *H. italicum*.

#### 2.2.7. *Helichrysum nudifolium*

##### Description and Ethnobotanical Usage

**Figure 9:**
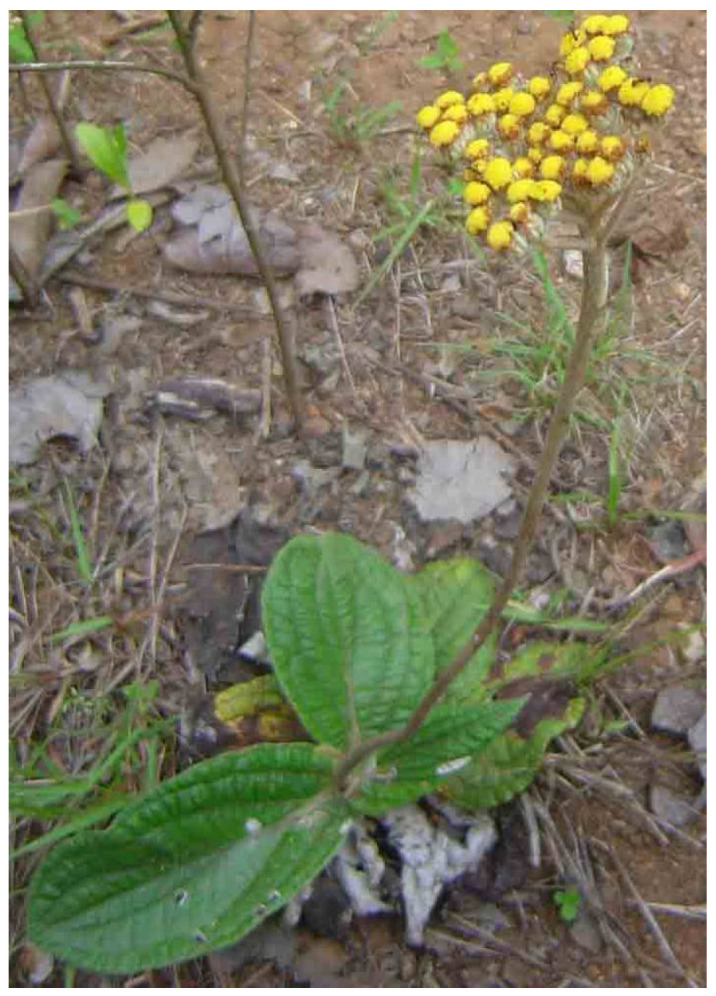
*H. nudifolium*. Source: Flora of Zimbabwe [67].

*Helichrysum nudifolium* (Figure 9) is a fast-growing plant with a light-yellow inflorescence and shiny green leaves. The plant’s flowering stalks can reach 1.5 m in height. It is very easy to grow in the garden and is widely found in South Africa. In South Africa, it is one of the most important species culturally, medicinally, and historically [65]. Traditionally, the fresh leaves or roots of *H. nudifolium* are boiled and taken orally for the treatment of diabetes [66]. Additionally, the leaves and roots are also used as traditional medicine for wound dressing, internal sores, and chest complaints [65].

##### Toxicity 

The study conducted by Lourens et al. [40] showed that the chloroform: methanol (1:1) extract of the plant displayed cytotoxicity activity with 73%, 83%, and 35% inhibitions, respectively, against transformed human kidney epithelial (Graham) cells, glioblastoma (SF-268) cells, and breast adenocarcinoma (MCF-7) at the tested concentration (0.1 mg/mL). Mokoka et al. [68], however, revealed that the whole plant dichloromethane:methanol (1:1) extract of *H. nudifolium* had low toxicity in rat myoblast L6 cells with a reported IC_50_ value of 47.7 µg/mL.

##### In Vitro Antidiabetic Study

The literature search revealed no reported in vitro antidiabetic activity of *H. nudifolium*.

##### In Vivo Antidiabetic Study

To date, there are no reported in vivo antidiabetic activities of *H. nudifolium*.

##### Antidiabetic activity of isolated compounds

Regrettably, there are no reports on the antidiabetic activity of the isolated compounds from *H. nudifolium*.

#### 2.2.8. *Helichrysum odoratissimum*

##### Description and Ethnobotanical Usage

**Figure 10:**
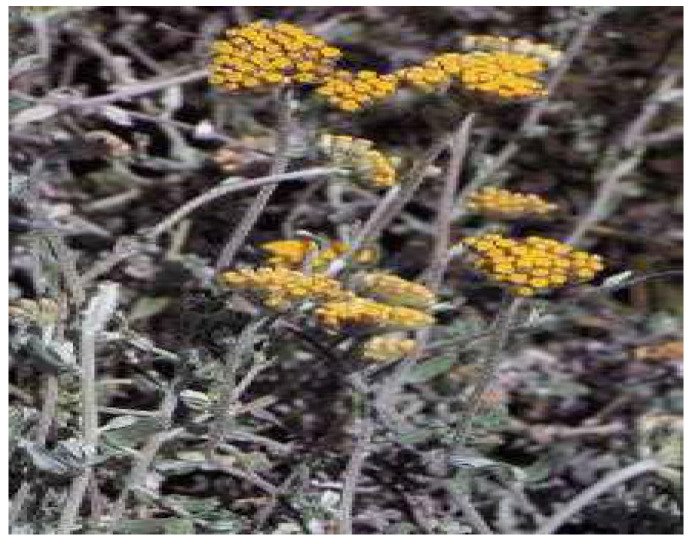
*H. odoratissimum*. Source: SANBI [70].

*Helichrysum odoratissimum* (Figure 10) is an aromatic, branched perennial plant with small grey leaves [69]. The leaves of the *H. odoratissimum* vary from linear-oblong, lingulate, to lanceolate. This plant has a yellow flowerhead borne in clusters at the tips of the twigs. *H. odoratissimum* is broadly found in South Africa, Mozambique, Zimbabwe, Lesotho, and Malawi [69]. In South Africa, it is found in the Eastern Cape across the mountains and coastal areas. In traditional medicine, the infusion from the whole plant is taken orally to treat diabetes [66]. In Lesotho, the whole plant part is mixed with other plants as herbal medicine to treat backache [69].

##### Toxicity

Studies on the toxicity of *H. odoratissimum* were documented by Lourens et al. [40] and Twilley et al. [71]. Lourens et al. [40] found the leaf and stem chloroform:methanol (1:1) extract of *H. odoratissimum* to be toxic against glioblastoma (SF-268) cells, transformed human kidney epithelial (Graham) cells, and breast adenocarcinoma (MCF-7) at 0.1 mg/mL, thereby displaying 48%, 17%, and 7.4% toxicity, respectively. While research conducted by Twilley et al. [71] revealed that the ethanol (100%) leaf and stem extract of *H. odoratissimum* exhibits toxicity against malignant melanoma (A 375), human embryonic kidney (HEK-293), human epidermoid carcinoma (A 431), and cervical epithelial carcinoma (HeLa) cell lines, with IC_50_ values at 55.5, 37.1, 33.1, and 15.5 µg/mL, respectively.

##### In Vitro Antidiabetic Study

Comprehensive search of the literature revealed no reports of in vitro antidiabetic activity of *H. odoratissimum*.

##### In Vivo Antidiabetic Study

The in vivo antidiabetic activity of the aqueous leaf extract of *H. odoratissimum* in alloxan-induced rats was demonstrated by Ngagi et al. [72]. The results indicated that the *H. odoratissimum* extract substantially lowered blood glucose levels in diabetic rats in a non-dose-dependent manner (between 50 to 150 mg/kg body weight).

##### Antidiabetic Activity of Isolated Compounds

To date, there are no reported studies involving the antidiabetic activity of the compounds from *H. odoratissimum*.

#### 2.2.9. *Helichrysum platicum*

##### Description and Ethnobotanical Usage

**Figure 11:**
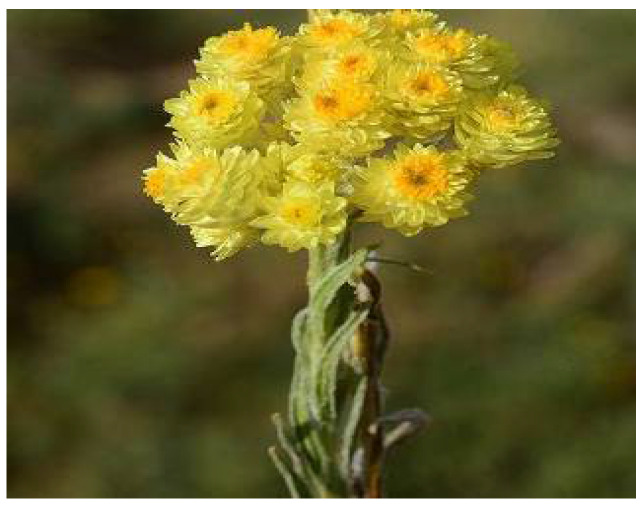
*H. plicatum*. Source: POWO [75].

*Helichrysum plicatum* (Figure 11) is a species belonging to the Helichrysum genus with simple and broad leaves. It is an herbaceous perennial plant that grows up to 0.24 m in height. *H. platicum* is widely found in Balkan, Iran, and Anatolian Peninsulas [73]. The infusion prepared from the plant is used to suppress diabetes symptoms [74].

##### Toxicity

Eroglu et al. [76] reported that the methanol (100%) flower extract of *H. platicum* exhibits toxicity properties in human lymphocytes at 0.5 mg/mL concentration. A separate study conducted by Bigović et al. [77] documented moderate toxicity of the ethanol (100%) and ethyl acetate: ethanol (100:0) flower extracts of the plant against human cervix adenocarcinoma cells (HeLa), prostate cancer (PC3) cells, and myelogenous leukemia (K562) cells, with IC_50_ values at 42.1 ± 0.05, 39.2 ± 1.1, and 25.9 ± 1.5 µg/mL, respectively.

##### In Vitro Antidiabetic Study

To the best of our knowledge, there are no reported of in vitro antidiabetic studies of *H. plicatum*.

##### In Vivo Antidiabetic Study

A research study indicated by Aslan et al. [78] revealed the in vivo antidiabetic activity of *H. plicatum* aqueous and ethanol extracts in normal and streptozotocin-induced diabetic rats. In the study, the results showed that the aqueous and ethanol extracts demonstrated significant antihyperglycemic activity at a concentration of 500 mg/kg body weight as compared with tolbutamide used as a positive control.

##### Antidiabetic Activity of Isolated Compounds

A comprehensive literature search showed that compounds such as isosalipurposide (102 mg), helichrysin A (87 mg), helichrysin B (220 mg), apigenin (300 mg), astragalin (28 mg), β-sitosterol (35 mg), β-sitosterol-3-*O*-β-d-glucopyranoside (25 mg), and nonacosanoic acid (15 mg), isolated from *H. platicum* methanol extract have been reported to exhibit alpha-glucosidase activity [79].

#### 2.2.10. *Helichrysum petiolare*

##### Description and Ethnobotanical Usage

**Figure 12:**
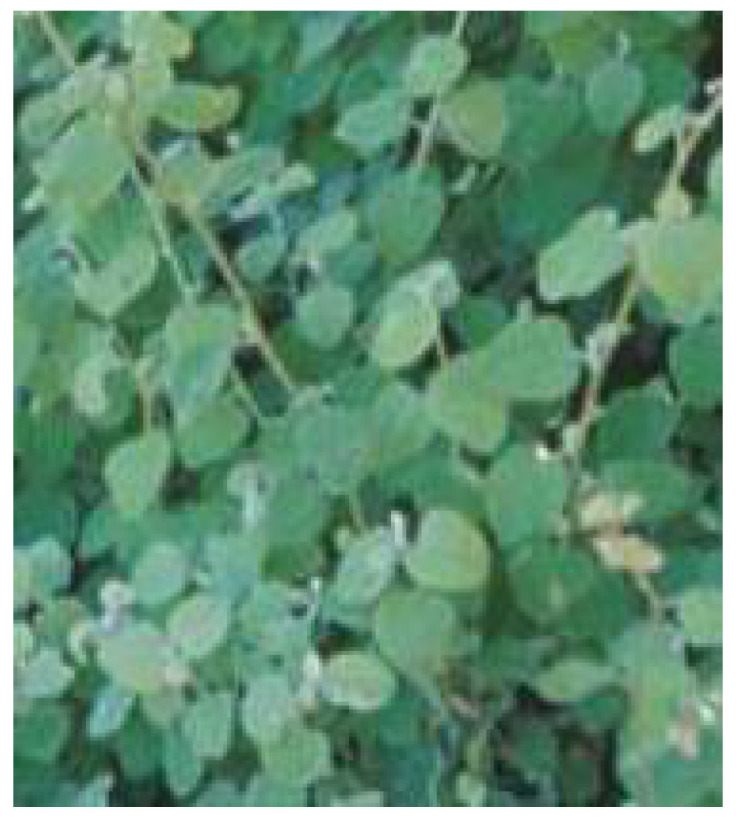
*H. petiolare*. Source: SANBI [83].

*Helichrysum petiolare* (Figure 12) is a vigorous shrub with silver-gray hair covering the aromatic round-shaped leaf [80]. It is one of the well-known and most used members of the *Helichrysum genus*. The plant grows to about 0.5 to 1 m in height with its flower whitish-cream in color. *H. petiolare* is found in the drier inland parts of South African provinces, such as the Eastern Cape and KwaZulu-Natal [81]. In South African traditional medicine, the infusion of the whole plant is taken orally to treat diabetes [66]. In addition, the decoction of the leaves of *H. petiolare* is used to improve skin texture and for wound healing [82].

##### Toxicity

An extensive search of the literature revealed at least three documented studies investigating the toxicity of *H*. *petiolare* [40,84,85]. Lourens et al. [40] reported that the chloroform:methanol (1:1) extract of *H. petiolare* had toxic effects on glioblastoma (SF-268), transformed human kidney epithelial (Graham), and breast adenocarcinoma (MCF-7) cells at 0.1 mg/mL, showing 76%, 59%, and 33% activity, respectively. The work of Aladejana et al. [84] revealed that the whole plant ethanol extract of *H. petiolare* demonstrated significant toxicity in L6 myocytes cells and HepG2 (C3A) hepatocytes at 100 μg/mL concentration. Sagbo and Otang-Mbeng [85] in their toxicity assessment of the methanol extract of *H petiolare* also reported that the extract was toxic against B16F10 mouse melanoma cells and MeWo human melanoma cells in a dose-dependent manner. The same group [85] also reported the genotoxicity of the plant extract (methanol) against the Vero cell line at the highest three concentrations tested (50, 100, and 200 µg/mL).

##### In Vitro Antidiabetic Study

The in vitro antidiabetic potential of *H. petiolare* using human hepatoma (HepG2/C3A) and rat skeletal (L6) myoblast cell lines has been shown [84]. The results of the study indicated that the whole plant boiled and cold aqueous extracts of *H. petiolare* significantly increased glucose uptake in L6 and HepG2/C3A cell lines at 25 µg/mL and 50 µg/mL, respectively. In the same study, it was also indicated that the extracts inhibited alpha-amylase and alpha-glucosidase activities in a dose-dependent manner as compared to the respective positive controls. In another study [86], the aqueous acetone extract of *H. petiolare* was shown to display an increased glucose uptake in HepG2 cells in a concentration-dependent manner and had moderate inhibitory effects against alpha-amylase and alpha-glucosidase activity compared to the acarbose, the positive control used in the study.

##### In Vivo Antidiabetic Study

To the best of our knowledge, there are no antidiabetic studies reported in vivo.

##### Antidiabetic Activity of Isolated Compounds

The compounds of *H. petiolare* displaying antidiabetic activity are yet to be reported.

**Table 1:** *Helichrysum* species used in the management of diabates mellitus.

S/N	*Helichrysum* Species	Plant Part Used	Mode of Preparation	Country Used for Diabetes	Reference
1	*Helichrysum arenarium* (L.) Moench	Aerial part	The aerial parts are used to make a decoction which is then taken orally	Turkey	[87]
2	*Helichrysum armenium* DC. subsp. Armenium	Aerial parts	The decoction from the aerial parts is then taken orally	Turkey	[88]
3	*Helichrysum aureum* (Houtt.) Merr.	Leaves	The crude (aqueous extract) is taken orally	South Africa, Mozambique, Zimbabwe, Lesotho, and Swaziland	[38]
4	*Helichrysum caespititium* (DC.) Harv.	Whole plant	The whole plant is cooked and then taken orally	South Africa	[43,89]
5	*Helichrysum chionophilum* Boiss. & Balansa	Unspecified	Unspecified	Turkey	[90]
6	*Helichrysum crispum* (L.) D. Don	Unspecified	The infusion is taken orally	South Africa	[91]
7	*Helichrysum cymosum* (L.) D. Don subsp. cymosum	Unspecified	Unspecified	South Africa	[92]
8	*Helicrysum devium* J.Y. Johnson	Unspecified	Unspecified	Portugal	[93]
9	*Helichrysum foetidum* (L.) Moench	Unspecified	Unspecified	South Africa	[9]
10	*Helichrysum graveolens* (Bieb.) Sweet	Capitulums	The capitlums decoction is taken orally	Anatolia, Turkey, and South Africa	[46]
11	*Helichrysum gymnocomum* DC var. acuminatum DC.DC.	Leaves	The leaves are used to make a decoction and then taken orally.	South Africa	[52]
12	*Helichrysum italicum* (Roth) G. Don	Unspecified	The infusion is taken orally	Turkey, Portugal, Italy, and Greece	[18,55]
13	*Helichrysum melaleucum* Rchb. Ex Holl	Unspecified	Unspecified	Portugal	[93]
14	*Helichrysum monizii* Lowe.	Unspecified	Unspecified	Portugal	[93]
15	*Helichrysum nudifolium* (L.) Less.	Leaves, roots	The decoction prepared from the leaves or roots is taken orally.	South Africa	[66]
16	*Helichrysum obconicum* DC.	Unspecified	Unspecified	Portugal	[93]
17	*Helichrysum odoratissimum* (L.) Sweet	Whole plant	The whole plant parts are used to make a decoction which is then taken orally	South Africa	[72]
18	*Helichrysum pallasii* (Sprengel) Ledeb	Leaf, Flower	The leaf or flower is used to make Infusion which is then taken orally	Turkey	[88]
19	*Helichrysum plicatum* DC.	Flower	The flower is used to make infusion where is then taken orally	Solhan, Anatolia, and Turkey	[74]
20	*Helichrysum petiolare* Hilliard & B.L. Burtt	Whole plant	The infusion prepared from the fresh plant is taken orally.	South Africa	[66]
21	*Helichrysum sanguineum* (L.) Kostel.	Unspecified	Unspecified	Palestine	[94]
22	*Helichrysum stoechas* (L.) *Moench*	Unspecified	Unspecified	Spain	[95]

**Table 2:** Reported antidiabetic activities of *Helichrysum* species.

S/N	Helichrysum Species	Plant Part Used	Extract	Antidiabetic Isolated Compounds	Toxicity	Antidiabetic Mechanism of Action	Model	Reference
1	*H. arenarium*	Flowers	Methanol	Isosalipurposide (**1**), aureusidin 6-*O*-β-d-glucopyranoside (**2**)	Toxic to Caco-2 and CCD112CoN at 1% *v*/*v* concentration	Inhibit Dipeptidyl peptidase-4 (DPP-4) activity and inhibitory effect against the increase in blood glucose levels in sucrose-loaded mice at 500 mg/kg concentration	In vitro and in vivo	[36,37]
2	*H. aureum*	*	*	*	Displayed cytotoxic effects toward Graham, MCF-7, and SF-268 cells at 0.1 mg/mL	*	*	[40]
3	*H. caespititium*	*	*	*	The dichloromethane extract has moderate toxicity toward H411E cell at 82.86 µg/mL concentration	*	*	[45]
4	*H. chionophilum*	Flowers and stem	Ethanol, methanol, and ethyl acetate	*	*	Inhibit alpha-glucosidase (between 3.77 to 25.42 mmol) and alpha-amylase (between 149.16 to 193.36 mmol) activities	In vitro	[90]
5	*H. cymosum* (L.) D. Don subsp. cymosum	Aerial parts	Methanol	Allopatuletin (**3**), dihydrobaicalein (**4**), helichrysetin (**5**)	Displayed cytotoxicity towards transformed human kidney epithelial cells at 17.47 µg/mL	Inhibit alpha-glucosidase activity between 14 to 44 µM concentrations	In vitro	[92,96]
6	*H. devium*	Leaves, flowers	Methanol	*	Toxic to Brin shrimp larvae between 2.36 to 4.85 µg/mL	Inhibit alpha-glucosidase (between 1.44 to 2.13 mg/mL) and alpha-amylase (between 1.85 to 2.39 mg/mL) activities	In vitro	[93,97]
9	*H. foetidum*	Leaves, flowers	Methanol	Helichrysetin (**5**)	Reported toxicity to Ha-CaT keratinocytes cells between 20 to 100 µg/mL	Inhibit alpha-glucosidase activity between 19.4 to 27.3 µg/mL	In vitro	[9]
10	*H. graveolens*	Capitulums	Ethanol, hydro-ethanolic and water	*	Not cytotoxic in Vero African green monkey kidney (up to 64 µg/mL) and C26 cells (up to 5.0 µg/mL)	Reduction of blood glucose levels in streptozotocin-induced diabetic rat (at 500 mg/kg), inhibition against alpha-glucosidase (at 0.7129 mg/mL), and alpha-amylase (at 3 mg/mL) activities	In vitro and in vivo	[46,48,49,50]
11	*H. italicum*	Flowers	Methanol-water	*	Toxic to U937 cell line (at 5% *v*/*v* concentration), Caco-2 cell line (at 1% *v*/*v* concentration), CCD112CoN cell line (at 2% *v*/*v* concentration), HeLa cell line (at 0.075 mg/mL), MCF-7 cell line (0.057 mg/mL), and B16F10 cell line	The inhibition against alpha-glucosidase (IC_50_ = 0.19 mg/mL) and alpha-amylase (IC_50_ = 0.83 mg/mL) activities and reduction of blood glucose levels in rats (at 2g/kg dose concentration)	In vitro and in vivo	[58,59,61]
12	*H. melaleucum*	Leaves, flowers	Methanol	*	Toxic to Brin shrimp larvae between 0.18 to 7.64 µg/mL concentrations	Inhibit alpha-glucosidase (between 0.99 to 0.125 mg/mL) and alpha-amylase (between 1.71 to 2.15 mg/mL) activities	In vitro	[92,97]
13	*H. monizii*	Aerial parts	Methanol	*	*	Inhibit alpha-glucosidase (at 2.76 mg/mL) and alpha-amylase (4.29 mg/mL) activities	In vitro	[93]
14	*H. nudifolium*	*	*	*	Exhibits cytotoxicity effects to Graham (at 0.1 mg/mL), SF-268 (at 0.1 mg/mL), MCF-7 (at 0.1 mg/mL), and rat myoblast L6 cells (IC_50_ = 47.7 µg/mL)	*	*	[40,68]
15	*H. obconicum*	Leaves	Methanol	*	Toxic to Brin shrimp larvae between 0.57 to 15.0 µg/mL concentrations	Inhibit alpha-glucosidase (at 1.35 mg/mL) and alpha-amylase (at 2.48 mg/mL) activities	In vitro	[93,97]
16	*H. odoratissimum*	Leaves	Aqueous	*	Shows toxicity to SF-268 (at 0.1 mg/mL), Graham (at 0.1 mg/mL), MCF-7 (at 0.1 mg/mL), A375 (IC_50_ = 55.5 µg/mL), HEK-293 (IC_50_ = 37.1 µg/mL), A431 (IC_50_ = 33.1 µg/mL), and HeLa (IC_50_ = 15.5 µg/mL) cells	Reduction of blood glucose level in the diabetes rat between 50 to 150 mg/kg dose concentration	In vivo	[40,71,72]
17	*H. plicatum*	Capitulums	Aqueous and ethanol	Isosalipurposide (**1**), helichrysin A (**6**), helichrysin B (**7**), apigenin (**8**), astragalin (**9**), β-sitosterol (**10**), β-sitosterol-3-glucoside (**11**), and nonacosanoic acid (**12**)	Exhibits toxicity effects against HeLa (IC_50_ = 42.1 µg/mL), PC3 (IC_50_ = 39.2 µg/mL), K562 (IC_50_ = 25.1 µg/mL), and human lymphocytes (at 0.5 mg/mL)	Reduction of blood glucose levels in streptozotocin-induced diabetic rat at 500 mg/mL dose concentration	In vivo	[76,78,79]
18	*H. petiolare*	Whole plant	Aqueous	*	Toxic to SF-268 (at 0.1 mg/mL), Graham (0.1 mg/mL), MCF-7 (at 0.1 mg/mL), HepG2 (C3A), L6 (at 100 µg/mL), B16F10 (between 25 to 100 µg/mL), MeWo (between 12.5 to 100 µg/mL), and Vero cells (between 50 to 200 µg/mL)	Enhance glucose uptake in L6 (at 25 µg/mL) and C3A (at 50 µg/mL) cell line	In vitro	[40,84,85]
19	*H. sanguineum*	Aerial parts	Aqueous	*	Reported to be toxic at high concentrations on human lymphocyte cells at 0.5 mg/mL concentration	Inhibit alpha-amylase activity with an IC_50_ = 28.1 µg/mL	In vitro	[94,98]
20	*H. stoechas*	Aerial parts	Methanol	*	Moderate toxicity was reported at the highest concentration (1 mg/mL) in HeLa cells	Inhibit alpha-amylase (between 0.46 to 0.63 mmol), alpha-glucosidase (IC_50_ = 481.0 µg/mL), and DPP-4 activity (IC_50_ = 81.7 µg/mL)	In vitro	[95,99]

* = Not available.

## 3. Material and Methods

A comprehensive literature survey was carefully conducted from August 2021 to February 2022. A report about the *Helichrysum* genus used traditionally in the management of diabetes was retrieved from various scientific databases such as Science Direct, Medline, Scopus, Web of Science, PubMed, Google Scholar, and Medline. In addition, ethnobotanical books, theses, and dissertations were also retrieved from various university libraries. The keywords and terms used during the search to obtain relevant articles or research papers were “*Helichrysum* species”, “diabetes”, “traditional medicine”, and “ethnopharmacology”.

## 4. Conclusions and Recommendations

The present study reviews the *Helichrysum* genus and its compounds’ activities in the management of diabetes mellitus. Out of the twenty-two *Helichrysum* species reported for the management of diabetes, only fifteen species have been scientifically evaluated, and many of these reported species exhibited their antidiabetic through inhibition of carbohydrate hydrolyzing enzymes (alpha-amylase and alpha-glucosidase) and reduction of blood glucose levels in streptozotocin-induced diabetic rats. The antidiabetic effects of these plants are attributed to several antidiabetic compounds, and only a few bioactive compounds have been identified in some species. However, it is worth noting that effort should be made to isolate more antidiabetic compounds from these species. In addition, an effort also needs to be devoted to the mechanism of antidiabetic action (in vitro and in vivo studies) of many previously explored and unexplored *Helichrysum* species.

## Data Availability

Not applicable.

## References

[1] Internal Diabets Federation (IDF) (2019). IDF Diabates Atlas. https://diabetesatlas.org/atlas/ninth-edition/.

[2] Galicia-Garcia U., Benito-Vicente A., Jebari S., Larrea-Sebal A., Siddiqi H., Uribe K.B., Ostolaza H., Martín C. (2020). Pathophysiology of Type 2 diabetes Mellitus. Int. J. Mol. Sci..

[3] Medscape Report of the Expert Committee on the Diagnosis and Classification of Diabetes Mellitus. https://www.medscape.com/viewarticle/412642_2.

[4] Mahmood N. (2016). A review of α-amylase inhibitors on weight loss and glycemic control in pathological state such as obesity and diabetes. Comp. Clin. Path..

[5] Pieme C.A., Tatangmo J.A., Simo G., Cabral P., Nya B., Jocelyne V., Moor A., Moukette B.M., Nzufo F.T., Legrand B. (2017). Relationship between hyperglycemia, antioxidant capacity and some enzymatic and non-enzymatic antioxidants in African patients with type 2 diabetes. BMC Res. Notes.

[6] Perkins R.M., Yuan C.M., Welch P. (2006). Dipsogenic diabetes insipidus: Report of a novel treatment strategy and literature review. Clin. Exp. Nephrol..

[7] Bedekar A., Shah K., Koffas M. (2010). Natural products for type II diabetes treatment. Adv. Appl. Microbiol..

[8] Osadebe P.O., Odoh E.U., Uzor P.F. (2014). Natural products as a potential sources of antidiabetic drugs. Br. J. Pharm. Res..

[9] Omolaja A.A., Pearce B., Omoruyi S.I., Badmus J.A., Ismail E., Marnewick J., Botha S., Benjeddou M., Ekpo O.E., Hussein A.A. (2021). The potential of chalcone-capped gold nanoparticles for the management of diabetes mellitus. Surf. Interfaces.

[10] Hung H., Qian K., Morris-natschke S.L., Hsu C., Lee K. (2012). Recent discovery of plant-derived anti-diabetic natural products. Nat. Prod. Rep..

[11] Rahmatullah M., Azam N.K., Khatun Z., Seraj S., Islam F., Rahman A., Jahan S., Aziz S. (2012). Medicinal plants used for treatment of diabetes by the Marakh Sect of the Garo Tribe living in Mymensingh district, Bangladesh. Afr. J. Tradit. Complement. Altern. Med..

[12] Ocvirk S., Kistler M., Khan S., Talukder S.H., Hauner H. (2013). Traditional medicinal plants used for the treatment of diabetes in rural and urban areas of Dhaka, Bangladesh—An ethnobotanical survey. J. Ethnobiol. Ethnomed..

[13] Odeyemi S., Greame B. (2018). Medicinal plants used for the traditional management of diabetes in the Eastern cape, South Africa:pharmacology and toxicology. Molecules.

[14] Kooti W., Farokhipour M., Asadzadeh Z., Ashtary-Larky D., Asadi-Samani M. (2016). The role of medicinal plants in the treatment of diabetes: A systematic review. Electron. Physician.

[15] Kasole R., Martin H.D., Kimiywe D. (2019). Traditional medicine and Its role in the management of Diabetes mellitus: “patients’ and herbalists’ perspectives”. Evid.-Based Complement. Altern. Med..

[16] Brusotti G., Cesari I., Dentamaro A., Caccialanza G., Massolini G. (2014). Isolation and characterization of bioactive compounds from plant resources: The role of analysis in the ethnopharmacological approach. J. Pharm. Biomed. Anal..

[17] Chinsembu K.C. (2019). Diabetes mellitus and nature’s pharmacy of putative antidiabetic plants. J. Herb. Med..

[18] Salehi B., Ata A., Anil Kumar N.V., Sharopov F., Ramírez-Alarcón K., Ruiz-Ortega A., Ayatollahi S.A., Fokou P.V.T., Kobarfard F., Zakaria Z.A. (2019). Antidiabetic potential of medicinal plants and their active components. Biomolecules.

[19] Duarte A.M., Guarino M.P., Barroso S., Gil M.M. (2020). Phytopharmacological strategies in the management of type 2 diabetes mellitus. Foods.

[20] IfedibaluChukwu E.I.M., Aparoop D., Kamaruz Z. (2020). Antidiabetic, anthelmintic and antioxidation properties of novel and new phyto compounds isolated from the methanolic stem-bark of *Vernonia amygdalina* Delile (Asteraceae). Sci. Afr..

[21] Hasan M., Uddin Q., Zaiton S., Soad M., Sarwar T. (2018). Animal models and natural products to investigate in vivo and in vitro antidiabetic activity. Biomed. Pharmacother..

[22] Ardalani H., Amiri F.H., Hadipanah A., Kongstad K.T. (2021). Potential antidiabetic phytochemicals in plant roots: A review of in vivo studies. J. Diabetes Metab. Disord..

[23] Akinyede K.A., Cupido C.N., Hughes G.D., Oguntibeju O.O. (2021). Medicinal properties and In vitro biological activities of selected *Helichrysum* species from South Africa: A review. Plants.

[24] Pooley E. (2003). Mountain Flowers: A Field Guide to the Flora of the Drakensberg and Lesotho.

[25] Antunes Viegas D., Palmeira-de-Oliveira A., Martinez-de-Oliveira J., Palmeira-de-Oliveira R. (2014). *Helichrysum italicum*: From traditional use to scientific data. J. Ethnopharmacol..

[26] Rigano D., Formisano C., Pagano E., Senatore F., Piacente S., Masullo M., Capasso R., Izzo A.A., Borrelli F. (2014). A new acetophenone derivative from flowers of *Helichrysum italicum* (Roth) Don ssp. italicum. Fitoterapia.

[27] Maroyi A. (2019). *Helichrysum caespititium* (DC.) Harv.: Review of its medicinal uses, phytochemistry and biological activities. J. Appl. Pharm. Sci..

[28] Tirillini B., Menghini L., Leporini L., Scanu N., Marino S., Pintore G. (2013). Antioxidant activity of methanol extract of *Helichrysum foetidum* Moench. Nat. Prod. Res..

[29] Matić I.Z., Aljančić I., Žižak Ž., Vajs V., Jadranin M., Milosavljević S., Juranić Z.D. (2013). In vitro antitumor actions of extracts from endemic plant *Helichrysum zivojinii*. BMC Complement. Altern. Med..

[30] Süzgeç-Selçuk S., Birteksöz A.S. (2011). Flavonoids of *Helichrysum chasmolycicum* and its antioxidant and antimicrobial activities. S. Afr. J. Bot..

[31] Ranaivoarisoa R.H., Ralambonirina S.T.R., Randriamialinor F., Randrianasolo R., Ratsimbason M., Ranarivelo L.R. (2020). Antiplasmodial Activity of the Extracts and Flavonoids Isolated from *Helichrysum gymnocephalum* Humbert (Asteraceae) from Madagascar. ACS Symp. Ser..

[32] Kherbache A., Senator A., Laouicha S., Al-Zoubi R.M., Bouriche H. (2020). Phytochemical analysis, antioxidant and anti-inflammatory activities of *Helichrysum stoechas* (L.) Moench extracts. Biocatal. Agric. Biotechnol..

[33] Pljevljakušić D., Bigović D., Janković T., Jelačić S., Šavikin K. (2018). Sandy everlasting (*Helichrysum arenarium* (L.) Moench): Botanical, chemical and biological properties. Front. Plant Sci.

[34] Shikov A.N., Pozharitskaya O.N., Makarov V.G., Wagner H., Verpoorte R., Heinrich M. (2014). Medicinal plants of the Russian Pharmacopoeia; their history and applications. J. Ethnopharmacol..

[35] POWO (2022). Plants of the World Online. https://powo.science.kew.org/taxon/urn:lsid:ipni.org:names:212418-1.

[36] Kramberger K., Pražnikar Z.J., Baruca Arbeiter A., Petelin A., Bandelj D., Kenig S. (2021). A comparative study of the antioxidative effects of *Helichrysum italicum* and *Helichrysum arenarium* infusions. Antioxidants.

[37] Morikawa T., Ninomiya K., Akaki J., Kakihara N., Kuramoto H., Matsumoto Y., Hayakawa T., Muraoka O., Wang L.-B., Wu L.-J. (2015). Dipeptidyl peptidase-IV inhibitory activity of dimeric dihydrochalcone glycosides from flowers of *Helichrysum arenarium*. J. Nat. Med..

[38] Balogun F.O., Tshabalala N.T., Ashafa A.O.T. (2016). Antidiabetic medicinal plants used by the Basotho Tribe of Eastern Free State: A Review. J. Diabetes Res..

[39] SANBI (South African National Biodiversity Institute) Red List of South African Plants. http://redlist.sanbi.org/species.php?species=3240-4002.

[40] Lourens A.C.U., Van Vuuren S.F., Viljoen A.M., Davids H., Van Heerden F.R. (2011). Antimicrobial activity and in vitro cytotoxicity of selected South African *Helichrysum* species. S. Afr. J. Bot..

[41] Meyer J.J.M., Lall N., Mathekga A.D.M., Jäger A.K. (2002). In vitro inhibition of drug-resistant and drug-sensitive strains of Mycobacterium tuberculosis by *Helichrysum caespititium*. S. Afr. J. Bot..

[42] Arnold T.H., Prentice C.A., Hawker L.C., Snyman E.E., Tomalin M., Crouch N.R., Pottas-Bircher C. (2002). Medicinal and Magical Plants of Southern Africa: An Annotated Checklist.

[43] Makhafola M., Middleton L., Olivier M., Olaokun O. (2019). Cytotoxic and antibacterial activity of selected medicinal. Asian J. Chem..

[44] Hyde M.A., Wursten B.T., Ballings P., Coates Palgrave M. Flora of Zimbabwe: Species Information: Individual Images: *Helichrysum caespititium*. https://www.zimbabweflora.co.zw/speciesdata/image-display.php?species_id=159510&image_id=5.

[45] Mamabolo M.P., Muganza F.M., Olivier M.T., Olaokun O.O., Nemutavhanani L.D. (2018). Evaluation of antigonorrhea activity and cytotoxicity of *Helichrysum caespititium* (DC) Harv. whole plant extracts. Biol. Med..

[46] Aslan M., Orhan D.D., Orhan N., Sezik E., Yeşilada E. (2007). A study of antidiabetic and antioxidant effects of *Helichrysum graveolens* Capitulums in Streptozotocin-Induced Diabetic Rats. J. Med. Food..

[47] POWO (2022). Plants of the World Online. https://powo.science.kew.org/taxon/urn:lsid:ipni.org:names:212825-1#bibliography.

[48] Kutluk I., Aslan M., Orhan I.E., Özçelik B. (2018). Antibacterial, antifungal and antiviral bioactivities of selected *Helichrysum* species. S. Afr. J. Bot..

[49] Yazdi T., Ehsan M., Amiri M.S., Akbari S., Sharifalhoseini M., Nourbakhsh F., Mashreghi M., Abbasi M.R., Modarres M., Es-haghi A. (2020). Green synthesis of silver nanoparticles using *Helichrysum graveolens* for biomedical applications and wastewater treatment. BioNanoScience.

[50] Orhan N., Hoçbaç S., Orhan D.D., Asian M., Ergun F. (2014). Enzyme inhibitory and radical scavenging effects of some antidiabetic plants of Turkey. Iran. J. Basic Med. Sci..

[51] Drewes S.F., van Vuuren S.E. (2008). Antimicrobial acylphloroglucinols and dibenzyloxy flavonoids from flowers of *Helichrysum gymnocomum*. Phytochemistry.

[52] Oyedemi S.O., Bradley G., Afolayan A.J. (2009). Ethnobotanical survey of medicinal plants used for the management of diabetes mellitus in the Nkonkobe municipality of South Africa. J. Med. Plant Res..

[53] FOSTER (The Friends of the St Francis Nature Areas). https://foster.org.za/plant-gallery-2/.

[54] Roussis V., Tsoukatou M., Petrakis P.V., Ioanna C., Skoula M., Harborne J.B. (2000). Volatile constituents of four *Helichrysum* species growing in Greece. Biochem. Syst. Ecol..

[55] Pereira C.G., Barreira L., Bijttebier S., Pieters L., Neves V., Rodrigues M.J., Rivas R., Varela J., Custódio L. (2017). Chemical profiling of infusions and decoctions of *Helichrysum italicum* subsp. picardii by UHPLC-PDA-MS and in vitro biological activities comparatively with green tea (*Camellia sinensis*) and rooibos tisane (*Aspalathus linearis*). J. Pharm. Biomed. Anal..

[56] American Botanical Council. https://www.herbalgram.org/resources/herbalgram/issues/105/table-of-contents/hg105-feat-helichrysum/.

[57] Kramberger K., Kenig S., Pražnikar Z.J., Glavač N.K., Barlič-Maganja D. (2021). A Review and Evaluation of the Data Supporting Internal Use of *Helichrysum italicum*. Plants.

[58] Staver M.M., Gobin I., Ratkaj I., Petrovic M., Vulinovic A., Dinarina-Sablic M., Broznic D. (2018). In vitro antiproliferative and antimicrobial activity of the essential oil from the flowers and leaves of *Helichrysum italicum* (Roth) G. Don growing in central Dalmatia (Croatia). J. Essent. Oil Bear. Plants.

[59] Gismondi A., Di Marco G., Canini A. (2020). *Helichrysum italicum* (Roth) G. Don essential oil: Composition and potential antineoplastic effect. S. Afr. J. Bot..

[60] Nostro A., Cannatelli M.A., Marino A., Picerno I., Pizzimenti F.C., Scoglio M.E., Spataro P. (2003). Evaluation of antiherpesvirus-1 and genotoxic activities of *Helichrysum italicum* extract. New Microbiol..

[61] de la Garza A.L., Etxeberria U., Lostao M.P., San Román B., Barrenetxe J., Martínez J.A., Milagro F.I. (2013). *Helichrysum* and grapefruit extracts inhibit carbohydrate digestion and absorption, improving postprandial glucose levels and hyperinsulinemia in Rats. J. Agric. Food Chem..

[62] Aćimović M., Ljujić J., Vulić J., Zheljazkov V.D., Pezo L., Varga A., Tumbas Šaponjac V. (2021). *Helichrysum italicum* (Roth) G. Don Essential Oil from Serbia: Chemical Composition, Classification and Biological Activity—May It Be a Suitable New Crop for Serbia?. Agronomy.

[63] de la Garza A.L., Etxeberria U., Palacios-Ortega S., Haslberger A.G., Aumueller E., Milagro F.I., Martínez J.A. (2014). Modulation of hyperglycemia and TNFα-mediated inflammation by *Helichrysum* and grapefruit extracts in diabetic db/db mice. Food Funct..

[64] de la Garza A.L., Etxeberria U., Haslberger A., Aumueller E., Martínez J.A., Milagro F.I. (2015). *Helichrysum* and grapefruit extracts boost weight loss in overweight rats reducing inflammation. J. Med. Food.

[65] Van Wyk B.E., Gorelik B. (2017). The history and ethnobotany of Cape herbal teas. S. Afr. J. Bot..

[66] Erasto P., Adebola P., Grierson D., Afolayan A.J. (2005). An ethnobotanical study of plants used for the treatment of diabetes in the Eastern Cape Province, South Africa. Afr. J. Biotechnol..

[67] Hyde M.A., Wursten B.T., Ballings P., Coates Palgrave M. Flora of Zimbabwe: Species Information: Individual Images: *Helichrysum nudifolium* var. *pilosellum*. https://www.zimbabweflora.co.zw/speciesdata/species.php?species_id=159810.

[68] Mokoka T.A., Zimmermann S., Julianti T., Hata Y., Moodley N., Cal M., Adams M., Kaiser M., Brun R., Koorbanally N. (2011). In vitro screening of traditional South African malaria remedies against *Trypanosoma brucei* rhodesiense, *Trypanosoma cruzi*, *Leishmania donovani*, and *Plasmodium falciparum*. Planta Med..

[69] Maroyi A. (2019). A synthesis and a review of medicinal uses, phytochemistry and biological activities of *Helichrysum odoratissimum* (L.) Sweet. Asian J. Pharm. Clin. Res..

[70] SANBI (South African National Biodiversity Institute) Helichrysum odoratissimum. http://pza.sanbi.org/Helichrysum-odoratissimum.

[71] Twilley D., Kishore N., Meyer D., Moodley I., Kumar V., Lall N. (2017). The effect of *Helichrysum odoratissimum* (L.) sweet on cancer cell proliferation and cytokine production. Int. J. Pharmacogn. Phytochem. Res..

[72] Ngagi J.M., Ngugi M.P., Kibiti C.M., Ngeranwa J., Njue W., Gathumbi P., Njabi E. (2015). Hypoglycemic effect of *Helichrysum odoratissimum* in alloxan induced diabetic mice. J. Pharmacol..

[73] Vujić B., Vidaković V., Jadranin M., Novaković I., Trifunović S., Tešević V., Mandić B. (2020). Composition, antioxidant potential, and antimicrobial activity of *Helichrysum plicatum* DC. various extracts. Plants.

[74] Polat R., Cakilcioglu U., Satıl F. (2013). Traditional uses of medicinal plants in Solhan (Bingöl—Turkey). J. Ethnopharmacol..

[75] POWO (2022). Plants of the World Online. https://powo.science.kew.org/taxon/urn:lsid:ipni.org:names:213189-1.

[76] Eroglu H.E., Budak Ü.M.I.T., Hamzaoglu E., Aksoy A., Albayrak S. (2010). In vitro cytotoxic effects of methanol extracts of six *Helichrysum* taxa used in traditional medicine. Pak. J. Bot..

[77] Bigović D., Šavikin K., Janković T., Menković N., Zdunić G., Stanojković T., Djurić Z. (2011). Antiradical and cytotoxic activity of different *Helichrysum plicatum* flower extracts. Nat. Prod. Commun..

[78] Aslan M., Orhan D.D., Orhan N., Sezik E., Yesilada E. (2007). In vivo antidiabetic and antioxidant potential of *Helichrysum plicatum* ssp. plicatum capitulums in streptozotocin-induced-diabetic rats. J. Ethnopharmacol..

[79] Aydin T. (2020). Secondary metabolites of *Helichrysum plicatum* DC. subsp. plicatum flowers as strong carbonic anhydrase, cholinesterase and α-glycosidase inhibitors. Z. Naturforsch. C.

[80] Deutschländer M.S., Lall N., van de Venter M. (2009). Plant species used in the treatment of diabetes by South African traditional healers: An inventory. Pharm. Biol..

[81] Serabele K., Chen W., Tankeu S., Combrinck S., Veale C.G., van Vuuren S., Chaudhary S.K., Viljoen A. (2021). Comparative chemical profiling and antimicrobial activity of two interchangeably used ‘Imphepho’species (*Helichrysum odoratissimum* and *Helichrysum petiolare*). S. Afr. J. Bot..

[82] Sagbo I., Mbeng W. (2018). Plants used for cosmetics in the Eastern Cape Province of South Africa: A case study of skin care. Pharmacogn. Rev..

[83] SANBI (South African National Biodiversity Institute) Helichrysum petiolare. http://opus.sanbi.org/bitstream/20.500.12143/3485/1/Helichrysumpetiolare_PlantzAfrica.pdf.

[84] Aladejana A.E., Bradley G., Afolayan A.J. (2021). In vitro evaluation of the anti-diabetic potential of *Helichrysum petiolare* Hilliard & B.L. Burtt using HepG2 (C3A) and L6 cell lines. F1000Research.

[85] Sagbo I.J., Otang-Mbeng W. (2020). Anti-proliferative and genotoxic activities of the *Helichrysum petiolare* Hilliard & BL Burtt. Sci. Pharm..

[86] Akinyede K.A., Oyewusi H.A., Hughes G.D., Ekpo O.E., Oguntibeju O.O. (2022). In Vitro Evaluation of the Anti-Diabetic Potential of Aqueous Acetone *Helichrysum petiolare* Extract (AAHPE) with Molecular Docking Relevance in Diabetes Mellitus. Molecules.

[87] Dalar A. (2018). Plant Taxa Used in the Treatment of Diabetes in Van Province, Turkey. Int. J. Pharm. Scond. Metab..

[88] Mükemre M., Behçet L., Çakılcıoğluc U. (2015). Ethnobotanical study on medicinal plants in villages of Çatak (Van-Turkey). J. Ethnopharmacol..

[89] Semenya S., Potgieter M., Erasmus L. (2012). Ethnobotanical survey of medicinal plants used by Bapedi healers to treat diabetes mellitus in the Limpopo Province, South Africa. J. Ethnopharmacol..

[90] Acet T., Ozcan K., Zengin G. (2020). An assessment of phenolic profiles, fatty acid compositions, and biological activities of two *Helichrysum* species: *Helichrysum plicatum* and *Helichrysum chionophilum*. J. Food Biochem..

[91] Hulley M.I., Van Vuuren S.F., Sadgrove N.J., Van Wyk B.-E. (2019). Antimicrobial activity of *Elytropappus rhinocerotis* (Asteraceae) against micro-organisms associated with foot odour and skin ailments. J. Ethnopharmacol..

[92] Jadalla B.M.I.S. (2020). Phytochemical and Biological Studies of *Helichrysum cymosum*. Master’s Thesis.

[93] Spínola V., Castilho P.C. (2017). Evaluation of Asteraceae herbal extracts in the management of diabetes and obesity. Contribution of caffeoylquinic acids on the inhibition of digestive enzymes activity and formation of advanced glycation end-products (in vitro). Phytochemistry.

[94] Jaradat N., Qneibi M., Hawash M., Sawalha A., Qtaishat S., Hussein F., Issa L. (2021). Chemical composition, antioxidant, antiobesity, and antidiabetic effects of *Helichrysum sanguineum* (L.) Kostel. from Palestine. Arab. J. Sci. Eng..

[95] Les F., Venditti A., Cásedas G., Frezza C., Guiso M., Sciubba F., Serafini M., Bianco A., Valero M.S., López V. (2017). Everlasting flower (*Helichrysum stoechas* Moench) as a potential source of bioactive molecules with antiproliferative, antioxidant, antidiabetic and neuroprotective properties. Ind. Crops Prod..

[96] Van Vuuren S.F., Viljoen A.M., van Zyl R.L., Van Heerden F.R., Başer K.H.C. (2006). The antimicrobial, antimalarial and toxicity profiles of helihumulone, leaf essential oil and extracts of *Helichrysum cymosum* (L.) D. Don subsp. cymosum. S. Afr. J. Bot..

[97] Gouveia-Figueira S.C., Gouveia C.A., Carvalho M.J., Rodrigues A.I., Nording M.L., Castilho P.C. (2014). Antioxidant capacity, cytotoxicity and antimycobacterial activity of madeira archipelago endemic *Helichrysum* dietary and medicinal plants. Antioxidants.

[98] Erolue E.H., Hamzaolu E., Aksoy A., Budak Ü., Özkul Y. (2010). In vitro genotoxic effects of four *Helichrysum* species in human lymphocytes cultures. Biol. Res..

[99] Zengin G., Cvetanović A., Gašić U., Tešić Z., Stupar A., Bulut B., Sinan K.I., Uysal S., Picot-Allain M.C.N., Mahomoodally M.F. (2020). A comparative exploration of the phytochemical profiles and bio-pharmaceutical potential of *Helichrysum stoechas* subsp. barrelieri extracts obtained via five extraction techniques. Process Biochem..

